# IL-12p40/IL-23p40 Blockade With Ustekinumab Decreases the Synovial Inflammatory Infiltrate Through Modulation of Multiple Signaling Pathways Including MAPK-ERK and Wnt

**DOI:** 10.3389/fimmu.2021.611656

**Published:** 2021-03-04

**Authors:** Renée H. Fiechter, Henriëtte M. de Jong, Leonieke J. J. van Mens, Inka A. Fluri, Sander W. Tas, Dominique L. P. Baeten, Nataliya G. Yeremenko, Marleen G. H. van de Sande

**Affiliations:** ^1^Amsterdam UMC, Department of Rheumatology and Clinical Immunology, Amsterdam Institute for Infection and Immunity, University of Amsterdam, Amsterdam, Netherlands; ^2^Amsterdam UMC, Department of Experimental Immunology, Amsterdam Institute for Infection and Immunity, University of Amsterdam, Amsterdam, Netherlands; ^3^Amsterdam Rheumatology and Immunology Center, Amsterdam, Netherlands

**Keywords:** psoriatc arthritis, synovium, spondyarthropathies, ustekinumab, PI3K - AKT pathway, MAPK pathway, Wnt pathway, IL-23/IL-17 axis

## Abstract

**Background:** Psoriatic arthritis (PsA) is a chronic inflammatory joint disease within the spondyloarthritis spectrum. IL-12p40/IL-23p40 blockade reduces PsA disease activity, but its impact on synovial inflammation remains unclear.

**Objectives:** To investigate the cellular and molecular pathways affected by IL-12p40/IL-23p40 blockade with ustekinumab in the synovium of PsA patients.

**Methods:** Eleven PsA patients with at least one inflamed knee or ankle joint were included in a 24-week single-center open-label study and received ustekinumab 45 mg/sc according to standard care at week 0, 4, and 16. Besides clinical outcomes, synovial tissue (ST) samples were obtained by needle arthroscopy from an inflamed knee or ankle joint at baseline, week 12 and 24 and analyzed by immunohistochemistry, RNA-sequencing and real-time quantitative polymerase chain reaction (qPCR).

**Results:** We obtained paired baseline and week 12, and paired baseline, week 12 and 24 ST samples from nine and six patients, respectively. Eight patients completed 24 weeks of clinical follow-up. At 12 weeks 6/11 patients met ACR20, 2/11 met ACR50 and 1/11 met ACR70 improvement criteria, at 24 weeks this was 3/8, 2/8 and 1/8 patients, respectively. Clinical and serological markers improved significantly. No serious adverse events occurred. We observed numerical decreases of all infiltrating cell subtypes at week 12, reaching statistical significance for CD68+ sublining macrophages. For some cell types this was even more pronounced at week 24, but clearly synovial inflammation was incompletely resolved. IL-17A and F, TNF, IL-6, IL-8, and IL-12p40 were not significantly downregulated in qPCR analysis of W12 total biopsies, only MMP3 and IL-23p19 were significantly decreased. RNA-seq analysis revealed 178 significantly differentially expressed genes between baseline and 12 weeks (FDR 0.1). Gene Ontology and KEGG terms enrichment analyses identified overrepresentation of biological processes as response to reactive oxygen species, chemotaxis, migration and angiogenesis as well as MAPK-ERK and PI3K-Akt signaling pathways among the downregulated genes and of Wnt signaling pathway among the upregulated genes. Furthermore, ACR20 responders and non-responders differed strikingly in gene expression profiles in a *post-hoc* exploratory analysis.

**Conclusions:** Ustekinumab suppresses PsA synovial inflammation through modulation of multiple signal transduction pathways, including MAPK-ERK, Wnt and potentially PI3K-Akt signaling rather than by directly impacting the IL-17 pathway.

## Introduction

Psoriatic arthritis (PsA) is a chronic inflammatory joint disease in the spondyloarthritis (SpA) spectrum, with an unknown etiology, high morbidity, and a great impact on quality of life. The IL-23/IL-17 and TNF pathways play major roles in PsA pathogenesis. IL-23 is composed of two subunits: the p19 subunit, specific for IL-23, and the p40 subunit, shared between IL-23 and IL-12 (1). IL12B, the gene encoding for the p40 subunit, and IL23R, the gene encoding its receptor, are both associated with PsA (2). Many biological treatments targeting the IL-23/IL-17 or TNF pathways decrease PsA activity (3). However, these treatments fail to induce remission in all patients, they vary in their effect on the different clinical PsA/SpA features (4–9), and although they effectively decrease inflammation and erosion development, they cannot halt new bone formation; underlining that there is still a need for new treatment strategies targeting all domains of SpA and inducing remission in all patients. Ustekinumab, a monoclonal antibody blocking the p40 subunit shared by IL-12 and IL-23, reduces PsA activity and has been shown to inhibit radiographic progression (4, 5, 10, 11), but how it affects synovial inflammation remains largely unknown.

The synovium is the key target tissue in inflammatory arthritis. Years of synovial tissue biopsy studies have greatly improved our understanding of SpA and PsA disease pathophysiology (12–14). Moreover, small focused mechanism of action trials with in depth analysis of synovial tissue responses before and after treatment further elucidated which pathophysiological processes are affected by specific treatments (15–17). This increased our understanding of pathways driving the chronic inflammatory response and showed which pathways could potentially be targeted to improve treatment responses and outcome in the future. We, therefore, set up this mechanism of action study, aimed to assess how IL-12p40/IL-23p40 blockade by ustekinumab impacts the cellular infiltrate and molecular pathways in the PsA synovium.

## Study Design

The Medical Ethics Committee of the Academic Medical Center Amsterdam (METC 2014_359; NL50218.018.14) approved this 24-week single-center open-label investigator-initiated mechanism of action study of ustekinumab (45 mg/sc at week 0, 4, and 16 according to standard care) which included eleven patients; all were clinically diagnosed with psoriatic arthritis, fulfilled the CASPAR classification criteria and gave written informed consent before enrollment.

### Patients

All patients had active disease with arthritis of at least a knee or an ankle joint. Patients were aged ≥18 years. Treatment with ustekinumab was indicated by the treating physician, but not initiated yet. A maximum of 50% of patients were allowed to have received TNFα inhibitors previously. Exclusion criteria were: contraindications for needle-arthroscopy such as joint replacement or the use of anticoagulation drugs, any therapy by intra-articular injections within 4 weeks before baseline, any intramuscular injection with corticosteroids within 2 weeks before baseline, and the use of an investigational drug or device within 4 weeks before baseline. Thirteen patients were screened for eligibility; eleven patients were included in this trial and participated up to and including the week 12 study visit. The needle-arthroscopy with synovial tissue biopsy sampling at 12 weeks was performed in nine patients. Due to anxiety, one patient declined another arthroscopy, but did complete all study visits. Another patient withdrew from the study after the week 12 study visit. Leading up to the 24 weeks study visit, two patients were excluded since intra-articular corticosteroids were given for persistent arthritis in a knee or finger joint. Technical difficulties prevented biopsy sampling in one patient at 24 weeks. In total, synovial tissue biopsy sampling was performed in six patients at 24 weeks, while eight patients were included in the efficacy analyses. All patients were males. The mean age and SD of our patients was 51.3 ± 11.1 years, their median symptom duration was 7 years [interquartile range (IQR) 2–14 years]. Several patients were on stable doses of concomitant medication at inclusion and remained so during the study: five (45.5%) patients used NSAIDs, one (9.1%) used oral corticosteroids (5 mg daily) and three (27.3%) used cDMARDs. Only two patients (18.2%) had previously been treated with a TNFα inhibitor.

### Clinical Assessments

Study visits were conducted at baseline and 4, 8, 12, and 24 weeks after baseline. Demographics, relevant medical history, and medication were recorded at baseline together with specific disease characteristics, such as duration of disease complaints, date of diagnosis, pattern of psoriatic arthritis complaints, and a family history of spondyloarthritis related diseases. At every visit, patients global disease activity and total pain on a Visual Analog Scale (VAS) of 0–100 mm, and the Bath Ankylosing Spondylitis Disease Activity Index (BASDAI) were recorded. We also recorded Physician global disease activity (VAS 0–100), 78/76 tender/swollen joint counts, enthesitis according to the Leeds Enthesitis Index (LEI), presence or absence of dactylitis per digit, Psoriasis Area and Severity Index (PASI) and the presence or absence of psoriatic nail dystrophy per digit. At every study visit, inflammatory markers C-reactive protein (CRP) and Erythrocyte Sedimentation Rate (ESR) were measured. Safety was assessed through routine safety lab, physical examination and registration of side effects. The primary efficacy endpoint was the number of patients meeting the American College of Rheumatology 20% improvement (ACR20) response at 12 weeks. Other clinical response outcome measures, such as ACR50, ACR70, European League Against Rheumatism (EULAR) Disease Activity Score (DAS) response and Psoriatic Arthritis Response Criteria (PsARC) response were assessed at both 12 and 24 weeks.

### Synovial Sampling by Arthroscopy

Synovial tissue biopsy samples were obtained by needle-arthroscopy from an inflamed knee or ankle at baseline and repeated in the same joint after 12 and 24 weeks, as described previously (18). Biopsy samples were immediately snap-frozen either en bloc in TissueTec (Sakura Finetek) for histologic evaluation or directly for RNA extraction (19).

### Immunohistochemical Staining

Cryostat sections (5 μm) were subsequently fixed with acetone, blocked with casein (Merck KGaA) and incubated with primary antibodies for 1 h at room temperature. Primary antibodies included CD3, LN10 (Vector laboratories); CD15, HI98 (Biolegend); CD55, 67 (Bio-connect); CD20, L26; CD68, EBM-11; CD138, MI15; CD163, Ber-MAC3 and vWF, polyclonal, A0082 (all Dako). Isotype- and concentration-matched antibodies were taken along as negative controls. After blocking for endogenous peroxidases, sections were incubated with horseradish-peroxidase-conjugated secondary antibodies [goat anti-mouse HRP, P0447 or swine anti-rabbit HRP, P0399 (both Dako)] for 30 min followed by staining with DAB chromogen (Biolegend). Two independent observers (NY and RF) scored the sections semiquantitatively on a 5-point scale, unaware of the corresponding patient and time point. CD68 was scored for lining and sublining separately.

### cDNA Library Generation and Next-Generation Sequencing

Total RNA was extracted from snap-frozen synovial biopsies using RNA Stat-60 (Tel-Test Inc), treated with DNase I (Invitrogen) and cleaned using RNeasy columns (79254, Qiagen) according to the manufacturer's instructions. RNA concentration and integrity were measured using Qubit RNA BR Assay kit (Life Technologies) and the Agilent 2100 Bioanalyzer (Agilent Technologies), correspondingly. cDNA libraries were constructed with Illumina TruSeq™ RNA Sample Preparation Kit (Illumina) using 1 μg of total RNA with RNA integrity number (RIN) ≥7. Briefly, the protocol consisted of polyA-RNA enrichment, RNA fragmentation, reverse transcription of fragmented RNA into cDNA, adapters ligation onto both ends of the cDNA fragments, and amplification of cDNA fragments by PCR. Resulting cDNA libraries were paired-end sequenced on Illumina NovaSeq 6000 by Macrogen (Seoul, Korea) to obtain around 60 million reads per sample. Macrogen then performed quality control of raw sequence reads (FastQC v0.11.7), removed low quality reads (Trimmomatic 0.38), mapped the reads to the reference genome (USCS hg19 assembly) with HISAT2 v2.1.0, assembled the transcript with Stringtie v1.3.4d, and calculated raw transcription profiles as a read count for each gene and each sample.

### Identification of Differentially Expressed Genes (DEGs)

We identified DEGs through count-based pipe-line edgeR (20); we analyzed and visualized DEGs data with edgeR, ggplot2, pheatmap, and enhanced Volcano packages using R v3.6.3 and RStudio v1.2.50 by BioBelka Genomics (Amsterdam). For pathway enrichment analysis, we used The Database for Annotation, Visualization and Integrated Discovery (DAVID) with default settings (21). We chose two to three genes from pathways and terms relating to pathophysiological processes and confirmed those by qPCR analysis.

### Real-Time Quantitative Polymerase Chain Reaction (qPCR)

Total RNA was reverse transcribed using a RevertAid H Minus First-Strand cDNA synthesis kit (Thermo Scientific) and analyzed by real-time qPCR with TaqMan gene expression assays for IL6 (Hs00174131_m1), IL8 (Hs00174103_m1), IL17A (Hs00174383_m1), IL17F (Hs00369400_m1), CCL20 (Hs00355476_m1), matrix metalloproteinase 3 (MMP3) (Hs00968305_m1), CXCL6 (Hs00605742_g1), TNFα (Hs00174128_m1), IL23p19 (Hs00372324_m1), IL12p40 (Hs01011518_m1), CD20 (Hs00544819_m1) IL12p35(Hs01073447), IL27p28(Hs00377366_m1), EBI3(Hs01057148_m1), FGF5 (Hs03676587_s1), DKK3 (Hs00247429_m1), WNT9a (Hs01573829_m1), FGF14 (Hs00738588_m1), PGF (Hs00182176_m1), NTF3 (Hs00267375_s1), CD70 (Hs00174297_m1), FOLR1 (Hs06631528_s1), VEGFC (Hs01099203_m1), CREB3L1 (Hs05025625_m1), SEMA5A (Hs01549381_m1), MUC1 (Hs00159357_m1), GLUL (Hs00365928_g1), ANGPTL1 (Hs00559786_m1), NOV (Hs00159631_m1), NOG (Hs00271352_s1), IER2 (Hs01109355_m1), AZIN1 (Hs00210634_m1) (Thermofisher) using Real-Time PCR QuantStudio3 PCR System (Applied Biosystems). Expressions of all genes were normalized to the expression of GAPDH (4310884E) as housekeeping gene and presented as fold change relative to the reference sample.

### Statistical Analysis

Clinical data are represented as median and interquartile range (IQR) unless mentioned otherwise. We analyzed clinical outcomes, real-time qPCR and immunohistochemistry using non-parametric statistics with the Wilcoxon matched pairs signed-rank test and considered values under 0.05 significant.

## Results

### Safety

During this study, 12 adverse events occurred, but no serious adverse events: eight patients had a common cold; one patient shortly experienced back, arm and leg pain after a bicycle ride; another had reoccurring arthritis of his unbiopsied knee for which he received intra-articular corticosteroids after his third arthroscopy; lastly, one patient reported headache and another stomach ache. Severe infections did not occur.

### Efficacy

The primary efficacy endpoint, ACR20 response at week 12, was reached in six out of 11 (54.5%) patients. Two (18.2%) patients achieved an ACR50 response and one (9.1%) patient an ACR70 response at 12 weeks. At week 24, three out of eight (37.5%) patients achieved an ACR20 response, two (25.0%) an ACR50 response and one (12.5%) an ACR70 response. PsARC response was achieved by two (18.2%) patients at week 12 and by three (37.5%) patients at week 24. Of the eleven patients, two (18.2%) patients had a good response according to the EULAR DAS response, three (27.3%) a moderate response and six (54.5%) were non-responders after 12 weeks of treatment. Of the eight patients at week 24, one (12.5%) patient had a good EULAR DAS response, two (25.0%) a moderate response and five (62.5%) were non-responders to treatment with ustekinumab. Table 1 shows changes in disease activity parameters in the separate domains, revealing that the improvement of psoriatic skin disease was most prominent. Two out of eleven (18.1%) patients had dactylitis at baseline, which changed to 1/11 (9.1%) and 0/8 (0.0%) patients after 12 and 24 weeks of ustekinumab treatment, respectively. Four out of eleven (36.4%) patients had enthesitis at baseline, which changed to 3/11 (27.3%) and 1/8 (12.5%) patients after 12 and 24 weeks of ustekinumab treatment, respectively.

**Table 1:** Disease activity parameters as observed for the total study population.

	**Baseline (*n* = 11)**	**Week 4 (*n* = 11)**	**Week 8 (*n* = 11)**	**Week 12 (*n* = 11)**	**Week 24 (*n* = 8)**	p*	p**
SJC76 (*n*)	2 (1–3)	1 (0–2)	1 (0–2)	2 (1–3)	2 (1–2)	0.394	0.715
TJC78 (*n*)	1 (0–5)	1 (0–4)	1 (1–2)	1 (1–2)	0 (0–1)	0.168	0.034
DAS28 (0–10)	2.8 (2.6–3.4)	2.2 (1.6–3.6)	1.7 (1.5–2.8)	2.0 (1.3–3.1)	2.0 (1.0–3.0)	0.026	0.036
PASI BSL>0 (0–72)	3.4 (1.8–15.7)^#^	2.4 (1.9–4.3)^#^	1.5 (0.5–2.0)^#^	0.8 (0–1.8)^#^	0 (0–4.1)^∧^	0.020	0.046
VAS patient global (0–100)	28 (16–55)	38 (23–68)	27 (10–63)	22 (12–46)	45 (3–51)	0.423	0.779
VAS patient pain (0–100)	16 (0–47)	22 (5–70)	19 (5–46)	17 (4–38)	12 (4–56)	0.241	0.888
BASDAI (0–10)	3.2 (2.4–4.7)	3.2 (1.5–5.7)	3 (1.5–4.6)	1.9 (1–3.8)	2.1 (0.4–4.2)	0.008	0.161
VAS physician (0–100)	48 (30–55)	34 (22–44)	20 (6–28)	15 (8–33)	8 (4–27)	0.007	0.021
CRP (mg/L)	8.5 (1.7–16.4)	2.3 (0.7–9.7)	2.2 (1.5–8.1)	1.6 (0.6–3.0)	1.0 (0.6–7.0)	0.008	0.012
ESR (mm/hr)	20 (8–35)	8 (5–28)	5 (2–20)	6 (2–17)	7 (2–16)	0.008	0.027

All values are presented as median (IQR). The p-value for the comparison of baseline and week 12 (*) or week 24 (**).

# *n = 9*,

∧ *n = 6*.

*SJC, swollen joint count; TJC, tender joint count; PASI, psoriasis area severity index; BSL, baseline; VAS, visual analog scale; BASDAI, Bath Ankylosing Spondylitis Disease Activity Index; CRP, C-reactive protein; ESR, erythrocyte sedimentation rate*.

### Immunomodulation of Synovial Inflammation by IL-12p40/IL-23p40 Blockade

**Figure 1:**
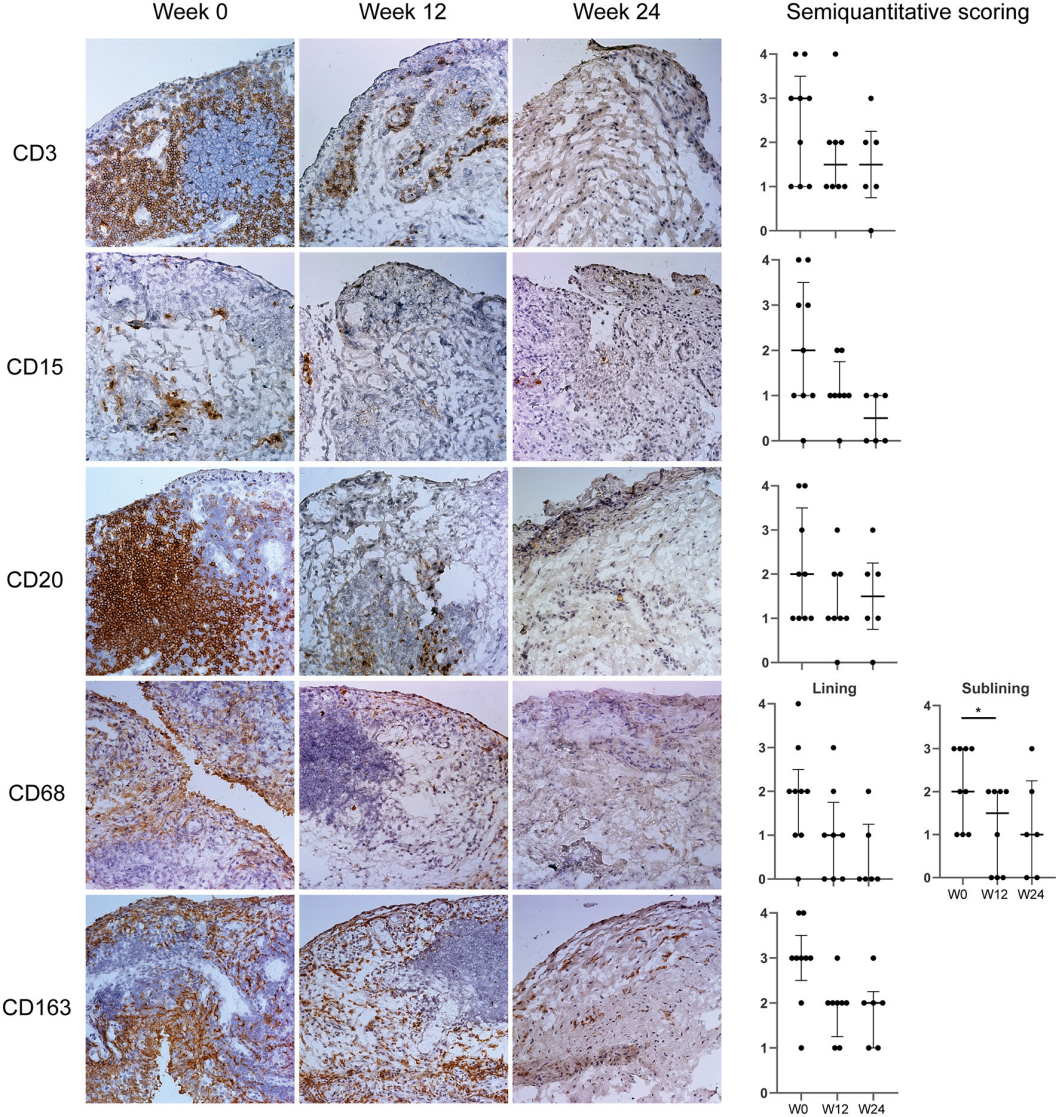
IL-12p40/IL-23p40 blockade by ustekinumab decreases the synovial infiltrate. **(Left)** Representative paired cryosections of synovial tissue obtained before (Week 0, W0, *n* = 9) and after 12 (W12, *n* = 8), and 24 (W24, *n* = 6) weeks of ustekinumab treatment, immunohistochemically stained for CD3 (T-cells), CD15 (neutrophils), CD20 (B-cells), CD68 (macrophages), and CD163 (alternatively activated macrophages). Original magnification x 20. **(Right)** Blind semiquantitative scoring. CD68 was scored for lining and sublining separately. Bars show median and interquartile ranges. Numerical decreases are seen for all cells, reaching significance for CD68 sublining macrophages after 12 weeks of ustekinumab treatment. *P < 0.05.

In order to assess whether IL-12p40/IL-23p40 blockade impacts synovial inflammation in PsA we examined immune cell infiltration before and after ustekinumab treatment by immunohistochemistry (Figure 1). We observed a numerical decrease of all infiltrating cell subtypes at week 12, except for CD138+ plasma cells, reaching statistical significance for CD68+ macrophages (*p* = 0.020) in the synovial sublining. This numerical decrease tended to be even more pronounced at week 24 for some cell types, but clearly ustekinumab treatment did not completely resolve the synovial inflammation. A small numerical decrease was found for von Willebrand factor (vWF)-positive endothelial cells and no differences were found for CD55+ cells present in the lining layer or CD138+ plasma cells (Supplementary Figure 1).

**Figure 2:**
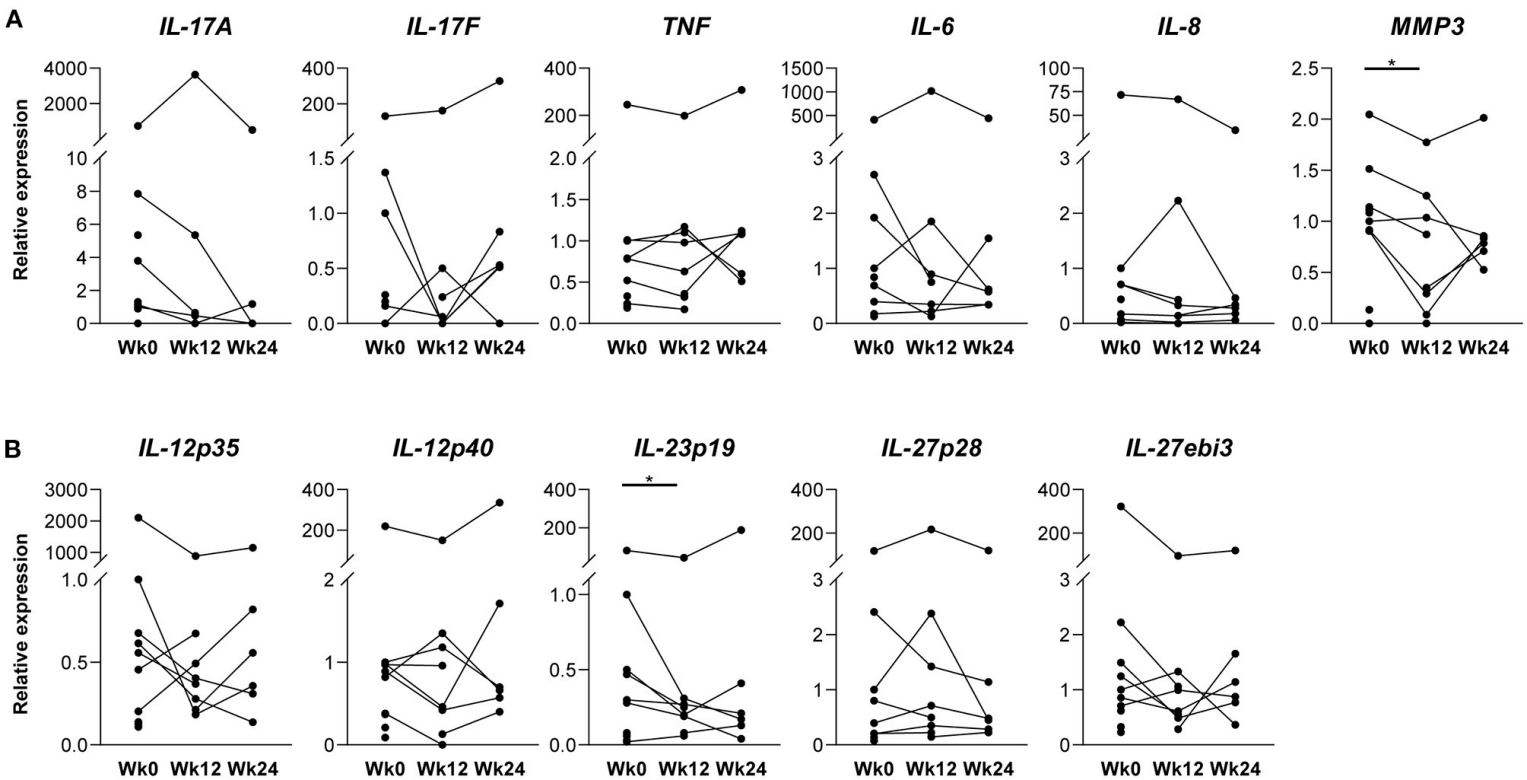
Key inflammatory cytokines and IL-12 family member subunits in synovial tissue before and after ustekinumab treatment. mRNA levels were assessed in total synovial biopsies before (W0, *n* = 10) and after 12 (W12, *n* = 8), and 24 weeks (W24, *n* = 6) of ustekinumab treatment by a real-time quantitative polymerase chain reaction, normalized to the expression of GAPDH housekeeping gene and presented as fold change relative to the reference sample. **(A)** Relative mRNA expression of interleukin (IL)-17A, IL-17F, tumor necrosis factor (TNF), IL-6 and IL-8 show no significant alterations after ustekinumab treatment, only matrix metalloproteinase 3 (MMP3) reaches borderline significance (*p* = 0.047) after 12 weeks of ustekinumab treatment. **(B)** Relative mRNA expression of IL-12p35, IL-12p40, IL-23p19, IL-27p28, and IL-27ebi3 show no effect of IL12-p40 blockade on mRNA expression of IL-12 family subunits, except for IL-23p19, which is significantly decreased after 12 weeks of ustekinumab treatment (*p* = 0.020). *P < 0.05.

Next, we investigated the expression of IL-17A and F, key cytokines downstream of IL-23, by real-time qPCR analysis of PsA synovium at the three time points and observed no significant alterations (Figure 2A). The expression of TNF, the other key pathogenic cytokine in PsA, also did not change. Looking further downstream, we found no significant differences for the pro-inflammatory cytokines IL-6 or IL-8, only mRNA levels of MMP3, showed a borderline significant decrease at week 12 (*p* = 0.047) (Figure 2A).

These findings show that despite the clear impact on inflammatory infiltration by histology, gene expression analysis failed to demonstrate any consistent and robust impact of p40 blockade by ustekinumab on the IL-23/IL-17 and TNF pathways.

### Effect of IL-12p40/IL-23p40 Blockade on IL-12 Family Members in the Inflamed PsA Synovial Tissue

IL-12 and IL-23 belong to the IL-12 cytokine family, which is postulated to have feedback mechanisms (1). We, therefore, assessed whether IL-12p40/IL-23p40 blockade impacts the expression of IL-12 family members. qPCR analysis revealed that the expression of most IL-12 family members, including IL-12p40 itself, did not significantly change at both time points (Figure 2B). However, mRNA expression of the IL23A gene—encoding IL-23p19, the subunit that heterodimerizes with IL-23p40 to form the IL-23 cytokine—did decrease significantly at week 12 as compared to baseline (*p* = 0.020), but not at week 24 of the treatment.

### Effect of IL-12p40/IL-23p40 Blockade on Whole PsA Synovial Tissue Transcriptome

**Figure 3:**
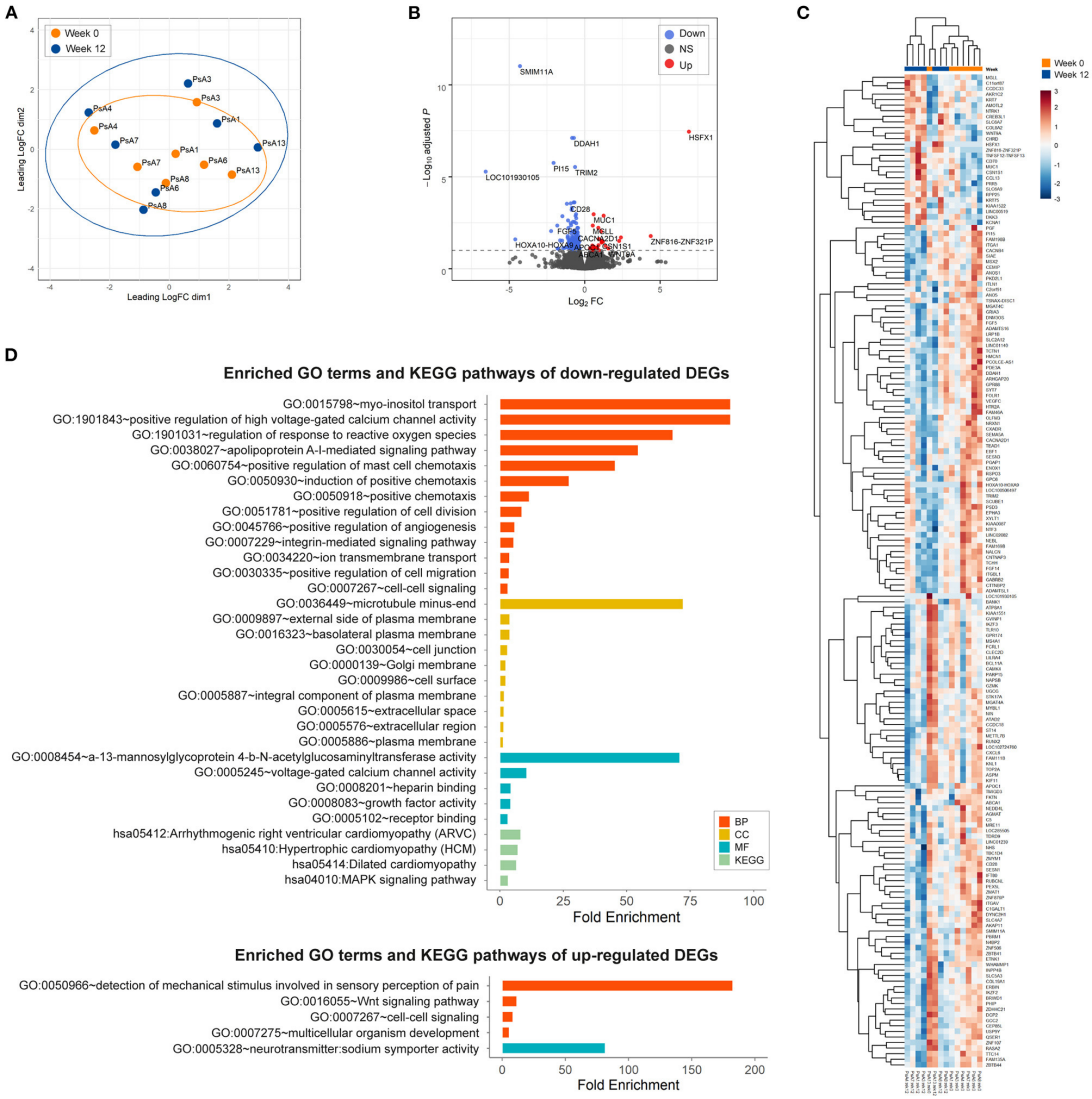
Differential gene expression and pathway analysis of changes in psoriatic arthritis synovium 12 weeks after treatment with ustekinumab. **(A)** Multidimensional scaling (MDS) plot of gene expression (logCPM) in pre-treatment (red, week 0, *n* = 7) and post-treatment (blue, week 12, *n* = 7) synovium reveals no distinct separation by the treatment effect. **(B)** A volcano plot identifying significantly differentially expressed genes (DEGs) in response to IL-23p40/IL-12p40 blockade with ustekinumab (FDR < 0.1, dashed line): red, upregulated genes; blue, downregulated genes; black, non-differentially expressed genes. **(C)** Hierarchical cluster analysis of all significant genes modulated by the treatment separates pre- and post-treated groups. Normalized scaled log2 gene expression levels are shown. **(D)** The enriched (*p* < 0.05) gene ontology (GO) terms and KEGG pathways of upregulated and downregulated DEGs in psoriatic arthritis synovium in response to the treatment with ustekinumab identified by DAVID. DEGs between pre- and post-treated groups were categorized into the three main categories of gene ontology classification: BP, biological process; CC, cellular component; MF, molecular function KEGG Kyoto Encyclopedia of Genes and Genomes.

To better understand the discrepancy between the histological improvement and the absence of impact on the IL-23/IL-17 and TNF pathways, and to investigate whether p40 blockade by ustekinumab affects other pathways in PsA synovitis, we performed an unbiased RNAseq analysis, on seven paired synovial biopsies obtained at baseline and after 12 weeks of treatment. A multidimensional scaling (MDS) plot based on 500 most variable genes failed to show a distinct separation between pre- and post-treatment groups, suggesting that biological variability between patients is larger than ustekinumab's treatment effect (Figure 3A). In total, 178 genes, including 27 upregulated and 151 downregulated genes, were identified as significantly differentially expressed in PsA synovium in response to the IL-12p40/IL-23p40 blockade using FDR cutoff 0.1 (Figure 3B and Table 2). Hierarchical clustering analysis of total differentially expressed genes (DEGs) revealed a better separation of pre- and post-treatment groups (Figure 3C). To gain more insight on biological processes modulated in PsA synovium in response to ustekinumab, we performed Gene Ontology (GO) terms and KEGG pathway enrichment analyses using the online software DAVID (https://david.ncifcrf.gov/). The downregulated DEGs were significantly enriched in Biological Processes (BP), such as voltage-dependent calcium channel activity (GO:1901843), myoinositol transport (GO:0015798), response to reactive oxygen species (GO:1901031), chemotaxis (GO:0050930; GO:0050918; GO:0060326; GO:0060754), migration (GO:0030335), positive regulation of endothelial cell proliferation (GO:0001938) and angiogenesis (GO:0001525); Cellular Components (CC), such as plasma membrane (GO:0009897; GO:0005887; GO:0005886), cell junctions (GO:0030054), and extracellular space (GO:0005615; GO:0005576); and in Molecular Functions (MF), such as growth factor activity (GO:0008083) and receptor binding (GO:0005102). In the KEGG pathway analysis, the downregulated DEGs were enriched in hypertrophic and dilated cardiomyopathy (hsa05410 and hsa05414), PI3K-Akt (hsa04151) and MAPK (hsa04010) signaling pathways (Figure 3D and Table 2). Wnt signaling (GO:0016055), cell-cell signaling (GO:0007267), and mechanical stimulus involved in sensory perception of pain (GO:0050966) GO terms were overrepresented within the upregulated DEGs. Ustekinumab thus seems to modulate both general inflammatory processes such as chemotaxis and angiogenesis, and specific molecular pathways such as MAPK and Wnt signaling, and potentially also PI3K-Akt signaling.

**Table 2:** Gene ontology and KEGG pathway enrichment analysis of differentially expressed genes upon IL12p40/IL23p40 blockade with ustekinumab.

**DEG class**	**Term**	**GO terms**	**DEG (total)**	***p* Value**	**FE**	**Genes**
**Up**						
	**GO BP**					
		GO:0050966~detection of mechanical stimulus involved in sensory perception of pain	2 (8)	0.010	182.5	KCNA1. NTRK1
		GO:0016055~Wnt signaling pathway	3 (187)	0.025	11.7	DKK3, WNT9A, AMOTL2
		GO:0007275~multicellular organism development	4 (521)	0.030	5.6	CHRD, DKK3, CREB3L1, WNT9A
		GO:0007267~cell-cell signaling	3 (254)	0.043	8.6	WNT9A, CCL13, CD70
		GO:0097191~extrinsic apoptotic signaling pathway	2 (42)	0.054	34.8	TNFSF12-TNFSF13, CD70
		GO:0030855~epithelial cell differentiation	2 (70)	0.088	20.9	MUC1, AKR1C2
		GO:0071300~cellular response to retinoic acid	2 (70)	0.088	20.9	WNT9A, MUC1
	**GO CC**					
		GO:0005615~extracellular space	8 (1347)	0.001	4.5	CHRD, DKK3, CSN1S1, WNT9A, CCL13, MUC1, TNFSF12-TNFSF13, CD70
		GO:0005887~integral component of plasma membrane	7 (1415)	0.007	3.8	KCNA1, MUC1, TNFSF12-TNFSF13, SLC6A9, SLC6A7, CD70, NTRK1
		GO:0031410~cytoplasmic vesicle	3 (235)	0.035	9.7	AMOTL2, KCNA1, NTRK1
		GO:0005576~extracellular region	6 (1610)	0.047	2.8	DKK3, CSN1S1, WNT9A, COL8A2, CCL13, TNFSF12-TNFSF13
		GO:0016324~apical plasma membrane	3 (291)	0.052	7.8	AMOTL2, KCNA1, MUC1
	**GO MF**					
		GO:0005328~neurotransmitter:sodium symporter activity	2 (18)	0.023	81.6	SLC6A9, SLC6A7
		GO:0005102~receptor binding	3 (353)	0.077	6.2	CCL13, TNFSF12-TNFSF13, CD70
**Down**						
	**GO BP**					
		GO:0050930~induction of positive chemotaxis	3 (15)	0.005	27.3	VEGFC, NTF3, PGF
		GO:0045766~positive regulation of angiogenesis	5 (115)	0.010	5.9	C5, SEMA5A, VEGFC, PGF, DDAH1
		GO:0034220~ion transmembrane transport	6 (210)	0.019	3.9	GABRB2, GRIA3, NEDD4L, NALCN, ANO5, ATP8A1
		GO:0015798~myo-inositol transport	2 (3)	0.022	91.0	PGAP1, SLC5A3
		GO:1901843~positive regulation of high voltage-gated calcium channel activity	2 (3)	0.022	91.0	FGF14, CACNA2D1
		GO:0050918~positive chemotaxis	3 (35)	0.027	11.7	SEMA5A, VEGFC, NTF3
		GO:1901031~regulation of response to reactive oxygen species	2 (4)	0.029	68.3	SESN1, SESN3
		GO:0007229~integrin-mediated signaling pathway	4 (99)	0.035	5.5	ITGAV, ITGBL1, ITGA1, ERBIN
		GO:0038027~apolipoprotein A-I-mediated signaling pathway	2 (5)	0.036	54.6	ITGAV, ABCA1
		GO:0007267~cell-cell signaling	6 (254)	0.038	3.2	FGF14, SEMA5A, CXCL6, NTF3, FGF5, PGF
		GO:0060754~positive regulation of mast cell chemotaxis	2 (6)	0.043	45.5	VEGFC, PGF
		GO:0030335~positive regulation of cell migration	5 (184)	0.045	3.7	ITGAV, SEMA5A, NTF3, CEMIP, ATP8A1
		GO:0051781~positive regulation of cell division	3 (47)	0.046	8.7	VEGFC, FGF5, PGF
		GO:0007155~cell adhesion	8 (459)	0.050	2.4	ANOS1, ITGAV, ITGBL1, ERBIN, SEMA5A, CNTNAP3, EPHA3, COL19A1
		GO:0008284~positive regulation of cell proliferation	8 (466)	0.054	2.3	HTR2A, RUNX2, ITGAV, VEGFC, PHIP, NTF3, FGF5, PGF
		GO:0070588~calcium ion transmembrane transport	4 (119)	0.056	4.6	ITGAV, CACNB4, PKD2L1, NALCN
		GO:0003073~regulation of systemic arterial blood pressure	2 (8)	0.057	34.1	ADAMTS16, DDAH1
		GO:1904262~negative regulation of TORC1 signaling	2 (9)	0.064	30.3	SESN1, SESN3
		GO:0050764~regulation of phagocytosis	2 (9)	0.064	30.3	ITGAV, CACNB4
		GO:0001525~angiogenesis	5 (223)	0.080	3.1	NRXN1, ITGAV, VEGFC, PGF, C1GALT1
		GO:0060326~cell chemotaxis	3 (65)	0.081	6.3	C5, SEMA5A, CXCL6
		GO:0001938~positive regulation of endothelial cell proliferation	3 (69)	0.090	5.9	SEMA5A, VEGFC, PGF
		GO:0010745~negative regulation of macrophage derived foam cell differentiation	2 (13)	0.090	21.0	ITGAV, ABCA1
		GO:0048010~vascular endothelial growth factor receptor signaling pathway	3 (72)	0.096	5.7	ITGAV, VEGFC, PGF
		GO:0033700~phospholipid efflux	2 (14)	0.097	19.5	ABCA1, APOC1
		GO:0007268~chemical synaptic transmission	5 (240)	0.098	2.8	HTR2A, NRXN1, GABRB2, CACNB4, GRIA3
		GO:0035725~sodium ion transmembrane transport	3 (73)	0.099	5.6	PKD2L1, SLC4A7, NALCN
		GO:0030054~cell junction	10 (459)	0.004	3.2	NRXN1, GABRB2, OLFM3, CXADR, ERBIN, PSD3, GRIA3, HMCN1, DCP2, CACNB4
		GO:0009897~external side of plasma membrane	6 (213)	0.016	4.1	ITGAV, ITGA1, ABCA1, MS4A1, CD28, SCUBE1
		GO:0005887~integral component of plasma membrane	18 (1415)	0.017	1.8	CLEC2D, NRXN1, FOLR1, GABRB2, TLR10, EPHA3, ABCA1, MS4A1, CD28, SLC5A3, HTR2A, ITGAV, CXADR, MYZAP, PRSS35, SLC4A7, GPR88, SLC2A12
		GO:0000139~Golgi membrane	10 (591)	0.021	2.4	FOLR1, FAM198B, ZDHHC21, UGCG, XYLT1, MGAT4A, MGAT4C, FKTN, ATP8A1, C1GALT1
		GO:0005886~plasma membrane	39 (4121)	0.027	1.4	CACNA2D1, CLEC2D, GABRB2, TLR10, CACNB4, GPR174, ABCA1, CD28, ATP8A1, HTR2A, FCRL1, CXADR, CNTNAP3, MYZAP, PREP0, PRSS35, NEDD4L, SLC4A7, IKZF3, ENOX1, NALCN, FOLR1, NRXN1, ANOS1, ITGA1, ERBIN, EPHA3, GRIA3, SLC5A3, CACNB4, CEMIP, DYNC2H1, ZDHHC21, ITGAV, SEMA5A, PKD2L1, ANO5, GPR88, SLC2A12
		GO:0036449~microtubule minus-end	2 (4)	0.027	72.3	NIN, ASPM
		GO:0009986~cell surface	9 (542)	0.033	2.4	FOLR1, CLEC2D, NRXN1, ITGAV, CXADR, ITGA1, CD28, PKD2L1, SCUBE1
		GO:0016323~basolateral plasma membrane	5 (180)	0.036	4.0	FOLR1, CXADR, ERBIN, PRSS35, SLC4A7
		GO:0005615~extracellular space	16 (1347)	0.043	1.7	ANOS1, MS4A1, SCUBE1, FGF5, SIAE, OLFM3, CXADR, C5, MYZAP, VEGFC, CXCL6, TCTN1, PRSS35, FKTN, ENOX1, PGF
		GO:0005576~extracellular region	18 (1610)	0.049	1.6	ANOS1, ITGBL1, XYLT1, EPHA3, APOC1, GZMK, FGF5, CEMIP, COL19A1, FGF14, CXADR, C5, CNTNAP3, VEGFC, CXCL6, NTF3, PGF, RSPO3
		GO:0043235~receptor complex	4 (127)	0.057	4.6	PEX5L, LRP1B, ITLN1, PKD2L1
		GO:0070062~extracellular exosome	27 (2811)	0.065	1.4	CACNA2D1, GABRB2, NEBL, HMCN1, ATAD2, ANAPC2, SIAE, DDAH1, ATP8A1, C5, ITLN1, PRSS35, NEDD4L, PHIP, MGAT4A, FOLR1, CAMK4, ITGA1, MS4A1, APOC1, CACNB4, DYNC2H1, AGMAT, KNL12, ITGAV, SEMA5A, PI15
		GO:0097431~mitotic spindle pole	2 (10)	0.067	28.9	NIN, ASPM
		GO:0061700~GATOR2 complex	2 (10)	0.067	28.9	SESN1, SESN3
	**GO MF**					
		GO:0005102~receptor binding	8 (353)	0.012	3.2	NRXN1, CXADR, C5, ITGA1, ERBIN, AOC1, NTF3, RSPO3
		GO:0008201~heparin binding	5 (160)	0.026	4.4	FGF14, ANOS1, CXCL6, PGF, RSPO3
		GO:0008083~growth factor activity	5 (162)	0.027	4.4	FGF14, VEGFC, NTF3, FGF5, PGF
		GO:0008454~alpha-1,3-mannosylglycoprotein 4-beta-N-acetylglucosaminyltransferase activity	2 (4)	0.028	70.9	MGAT4A, MGAT4C
		GO:0005245~voltage-gated calcium channel activity	3 (40)	0.032	10.6	CACNA2D1, ITGAV, CACNB4
		GO:0046872~metal ion binding	22 (2069)	0.050	1.5	CACNA2D1, NRXN1, ITGA1, EBF1, ZMAT1, RASA2, BCL11A, ZBTB41, DDAH1, AGMAT, ZNF876P, ITGAV, ZNF506, ZBTB44, PDE3A, MGAT4A, MGAT4C, IKZF3, IKZF2, ZNF107, TRIM2, C1GALT1
		GO:0008509~anion transmembrane transporter activity	2 (9)	0.061	31.5	AOC1, SLC4A7
		GO:0003676~nucleic acid binding	12 (985)	0.084	1.7	CLEC2D, TTC14, ZMYM1, ZNF506, ZBTB44, BCL11A, TDRD9, ZBTB41, ENOX1, IKZF3, IKZF2, ZNF107
		GO:0001618~virus receptor activity	3 (70)	0.086	6.1	HTR2A, ITGAV, CXADR
		GO:0008144~drug binding	3 (76)	0.099	5.6	FOLR1, HTR2A, TOP2A
		GO:0005272~sodium channel activity	2 (15)	0.100	18.9	PKD2L1, NALCN
	**KEGG**					
		hsa05412:Arrhythmogenic right ventricular cardiomyopathy (ARVC)	4 (67)	0.011	8.4	CACNA2D1, ITGAV, ITGA1, CACNB4
		hsa05410:Hypertrophic cardiomyopathy (HCM)	4 (78)	0.017	7.2	CACNA2D1, ITGAV, ITGA1, CACNB4
		hsa05414:Dilated cardiomyopathy	4 (84)	0.020	6.7	CACNA2D1, ITGAV, ITGA1, CACNB4
		hsa04010:MAPK signaling pathway	6 (253)	0.031	3.3	FGF14, CACNA2D1, CACNB4, RASA2, NTF3, FGF5
		hsa04014:Ras signaling pathway	5 (226)	0.072	3.1	FGF14, VEGFC, RASA2, FGF5, PGF
		hsa04151:PI3K-Akt signaling pathway	6 (345)	0.091	2.4	FGF14, ITGAV, ITGA1, VEGFC, FGF5, PGF

*Twenty seven significantly upregulated and 151 significantly downregulated genes in response to the treatment with ustekinumab (FDR 0.1) were imported into DAVID database. GO terms and KEGG pathway analysis were performed to identify biological processes significantly enriched (p < 0.05) among anti- IL12p40/IL23p40 regulated genes in the synovium of patients with PsA. DEG, differentially expressed genes; FE, fold enrichment; GO BP, gene ontology term biological process; GO CC, gene ontology term cellular component; GO MF, gene ontology term molecular function; KEGG, Kyoto Encyclopedia of Genes and Genomes*.

**Figure 4:**
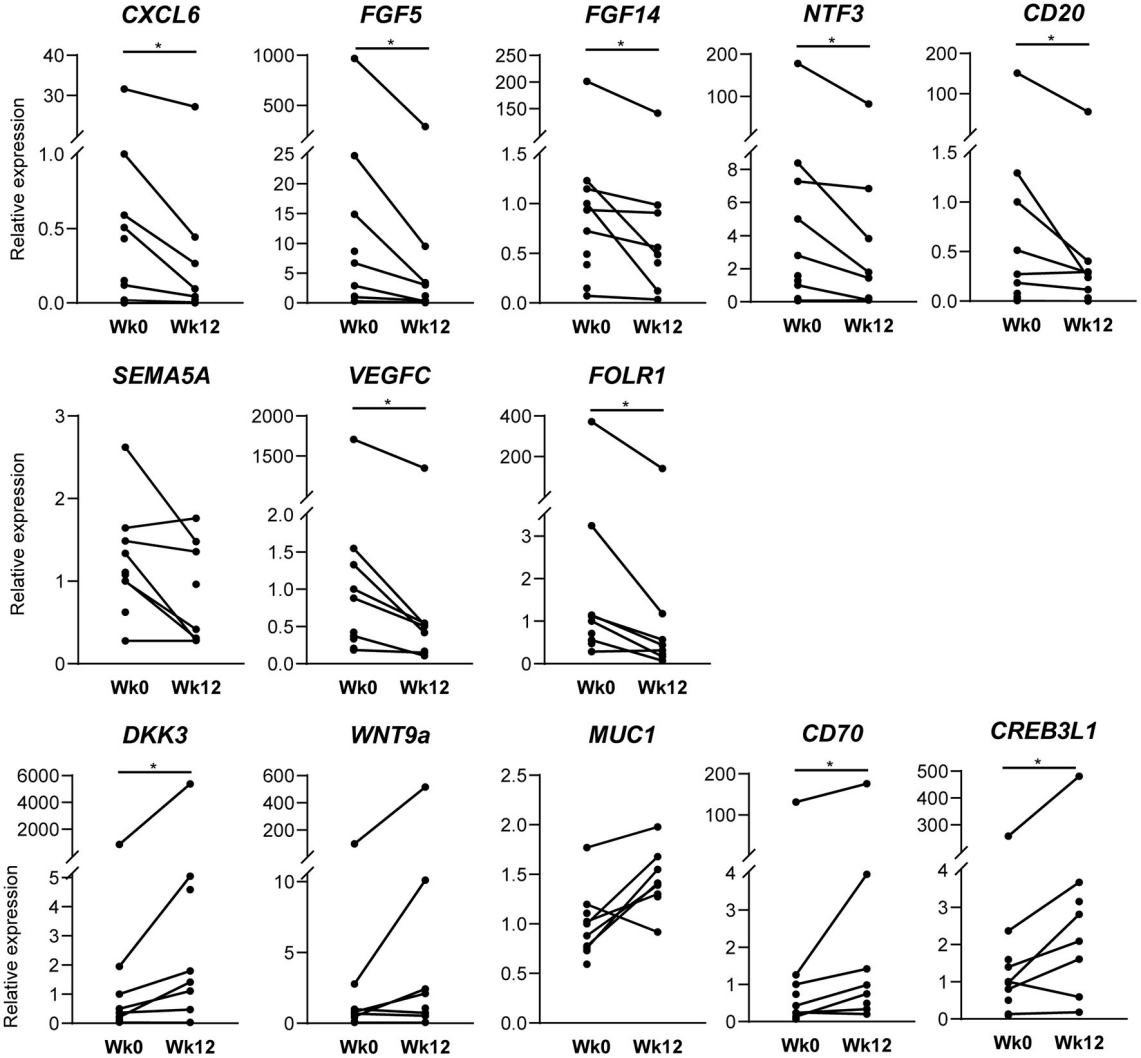
Confirmation of RNA sequencing analysis by qPCR. Real-time quantitative polymerase chain reaction analysis of synovial tissue of baseline (*n* = 10) and after 12 weeks (*n* = 8) of ustekinumab treatment confirms the differential expression of genes found in RNA sequencing: CD20, CXCL6, FGF5, fibroblast growth factor 5; FGF14; FOLR1, folate receptor 1; NTF3, neurotrophin 3; SEMA5A, semaphorin 5A; VEGFC, vascular endothelial growth factor C for downregulated DEGs; CD70, CREB3L1, CAMP Responsive Element Binding Protein 3 Like 1; DKK3, Dickkopf WNT Signaling Pathway Inhibitor 3; MUC1, mucin 1; WNT9A, WNT family member 9A for upregulated genes *p < 0.05.

To validate the results of the RNA sequencing and pathway analysis, we determined expression levels of selected genes by qPCR at baseline and after 12 weeks of the treatment. qPCR analysis confirmed changes in the expression levels for most of the selected DEGs resulted from the RNAseq analysis: CXCL6 (*p* = 0.016), FGF5 (*p* = 0.016), FGF14 (*p* = 0.016), NTF3 (*p* = 0.031), CD20 (*p* = 0.046), SEMA5A (*p* = 0.078), VEGFC (*p* = 0.016), FOLR1 (*p* = 0.031) for downregulated DEGs; DKK3 (*p* = 0.030), WNT9A (*p* = 0.078), MUC1 (*p* = 0.078), CD70 (*p* = 0.031), and CREB3L1 (*p* = 0.047) for upregulated DEGs (Figure 4).

### ACR20 Responders and Non-responders Differ in Gene Expression Profiles

**Figure 5:**
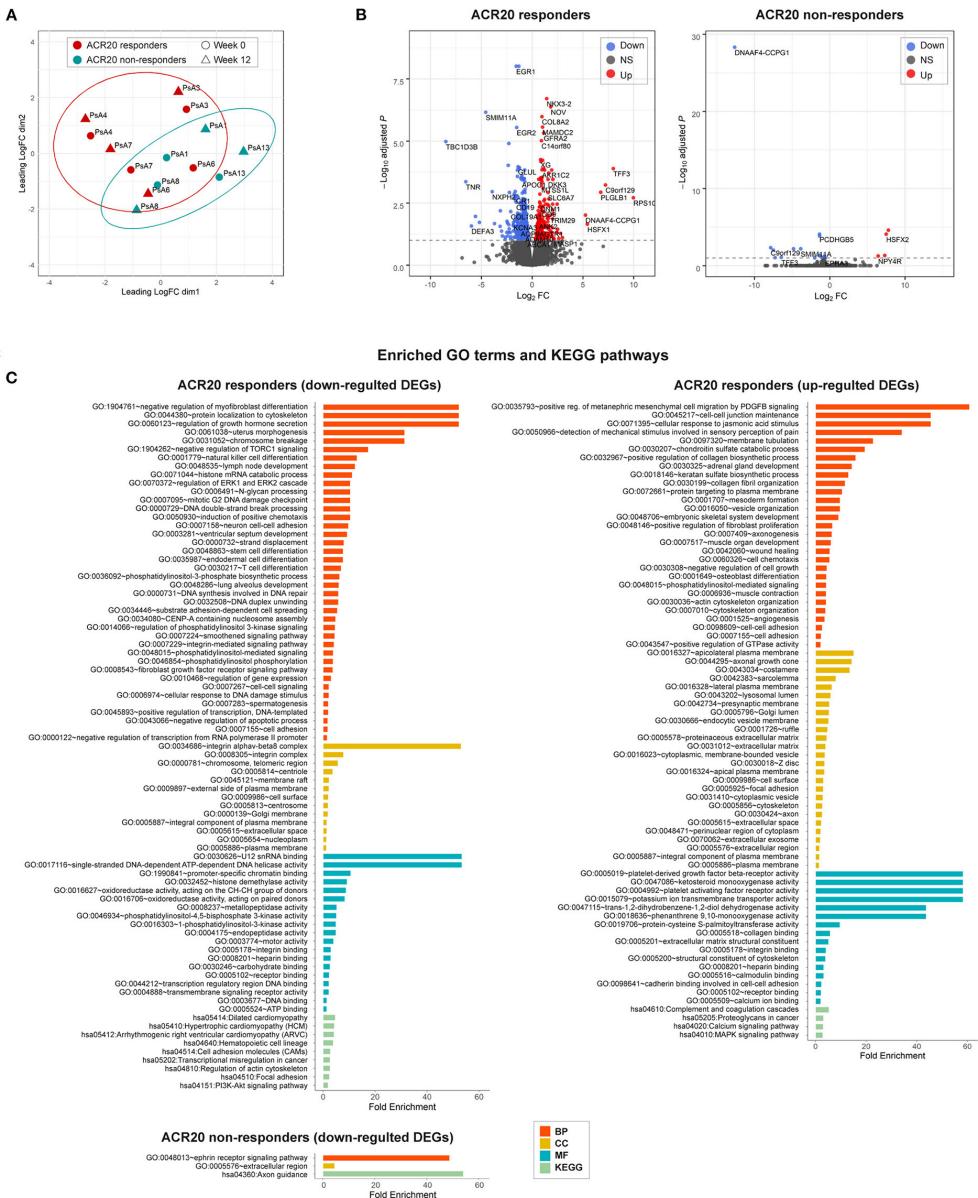
Differential gene expression and pathway analysis of ACR20 responders and non-responders to ustekinumab treatment in a *post-hoc* explorational analysis. **(A)** Multidimensional scaling (MDS) plot of gene expression (logCPM) in American College of Rheumatology 20% improvement criteria (ACR20) responders (red, *n* = 4) and ACR20 non-responders (blue, *n* = 3) reveals separation of the two groups. **(B)** A volcano plot identifying significantly differentially expressed genes (DEGs) of ACR20 responders and non-responders to IL-23p40/IL-12p40 blockade with ustekinumab (FDR < 0.1, dashed line): red, upregulated genes; blue, downregulated genes; black, non-differentially expressed genes. **(C)** The enriched (*p* < 0.05) gene ontology (GO) terms and KEGG pathways of upregulated and downregulated DEGs in psoriatic arthritis synovium in response to the treatment with ustekinumab identified by DAVID for ACR20 responders and non-responders. DEGs between pre- and post-treated groups were categorized into the three main categories of gene ontology classification: BP, biological process; CC, cellular component; MF, molecular function.

**Figure 6:**
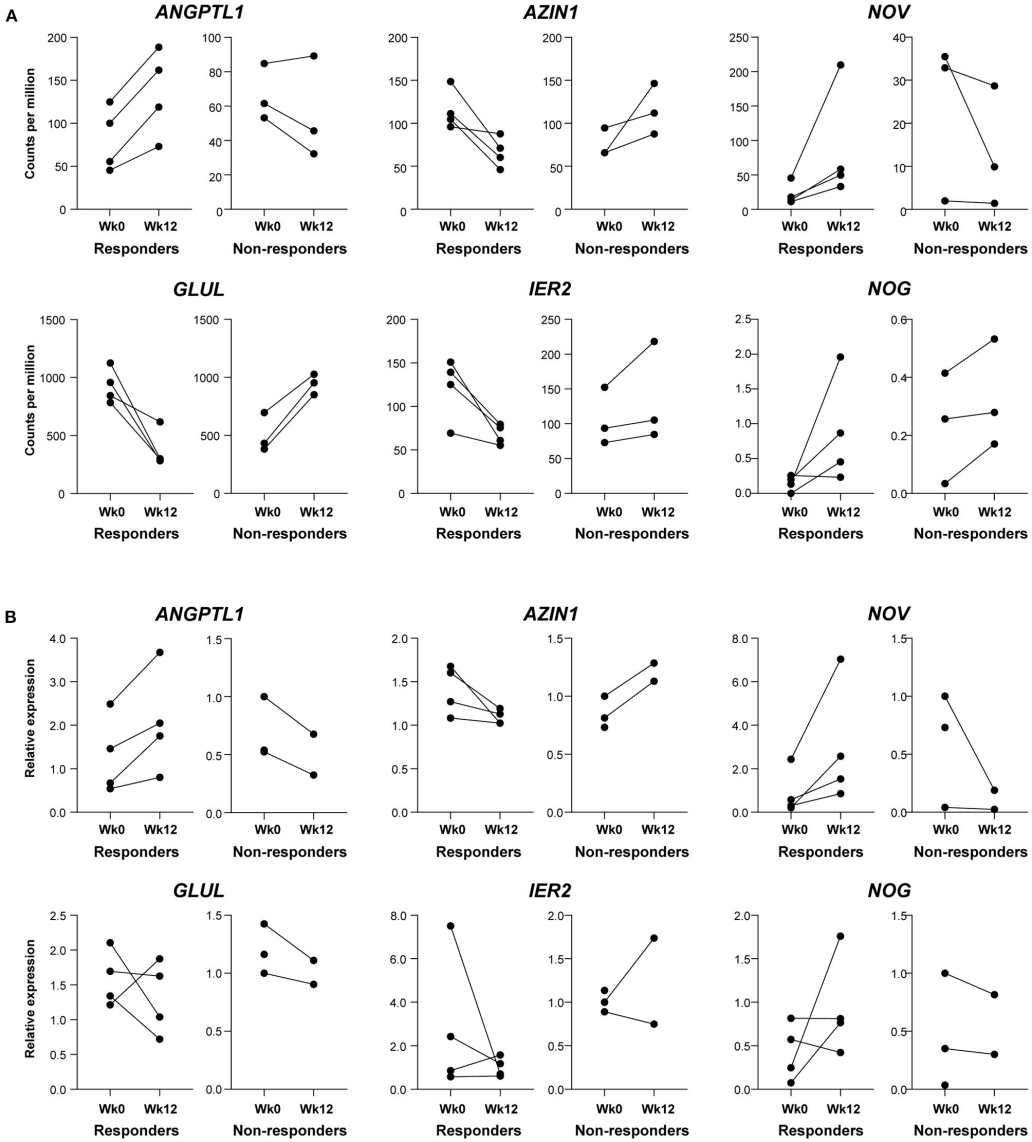
Confirmation of difference in gene expression profiles of ACR20 responders and non-responders by qPCR. **(A)** Genes responding significantly different to ustekinumab treatment between ACR20 responders (*n* = 4) and non-responders (*n* = 3) in RNAseq analysis that were selected for validation. **(B)** Real-time quantitative polymerase chain reaction analysis of synovial tissue of baseline and after 12 weeks of ustekinumab treatment for angiopoietin-like 1 (ANGPTL1), antizyme inhibitor 1 (AZIN1), glutamine synthetase (GLUL), immediate early response 2 (IER2), noggin (NOG), and nephroblastoma overexpressed (NOV) confirm that ANGPTL1, AZIN1, and NOV levels go in opposite directions for ACR20 responders and non-responders.

Strikingly, a *post-hoc* exploratory analysis of RNAseq data revealed a marked difference in gene expression profiles between ACR20 responders and non-responders. MDS plot based on 500 most variable genes shows a noticeable separation of ACR20 responders and non-responders groups (Figure 5A). The number of DEGs in the ACR20 non-responders group was very low, comprising only 19 DEGs, while the number of DEGs in the ACR20 responders group was increased compared to the total group, comprising 632 vs. 178 DEGs respectively (Figure 5B). Accordingly, only a few GO terms and KEGG pathways were enriched in downregulated DEGs of ACR20 non-responders, such as ephrin receptor signaling pathway (GO:0048013) and axon guidance (hsa04360); while numerous terms and pathways were enriched in the DEGs of ACR20 responders, including negative regulation of myofibroblast differentiation (GO:1904761), regulation of ERK1 and ERK2 cascade (GO:0070372) and PI3K-Akt signaling (hsa04151) for downregulated DEGs and collagen fibril organization (GO:0030199), osteoblast differentiation (GO:0001649), positive regulation of protein kinase B signaling (GO:0051897), positive regulation of fibroblast proliferation (GO:0048146) and complement and coagulation cascades (hsa04610) for upregulated DEGs (Figure 5C). To confirm the in RNAseq analysis observed differences between ACR20 responders and non-responders, we assessed the expression of genes that responded significantly different to ustekinumab treatment between both groups (Figure 6A). qPCR analysis confirmed that expressions of angiopoietin-like 1 (ANGPTL1), antizyme inhibitor 1 (AZIN1) and nephroblastoma overexpressed (NOV) changed in opposite directions for ACR20 responders and non-responders in response to the treatment (Figure 6B). Changes in expression of glutamine synthetase (GLUL), immediate early response 2 (IER2) and noggin (NOG) were less clear in the qPCR analysis as compared to the RNAseq data (Figure 6B). Similarly to the total group the PI3K-Akt-mTOR and MAPK-ERK signaling pathways were modulated in the ACR20 responders. However, in contrast to the total group, in the ACR20 responders p40 targeting with ustekinumab resulted in modulation of specific remodeling pathways such as myofibroblast and osteoblast differentiation and fibroblast proliferation.

## Discussion

To advance our knowledge of PsA pathophysiology and disease management, we set up this mechanism of action study, aimed to assess how IL-12p40/IL-23p40 blockade by ustekinumab impacts the cellular infiltrate and molecular pathways in the PsA synovium.

Within the limitations of this mechanistic trial design, we observed that of our PsA patients 55 percent reached the primary efficacy outcome, an ACR20 response after 12 weeks of ustekinumab treatment. This aligns with previous PsA studies with ustekinumab (4, 5) and thus enables us to study the impact of ustekinumab on infiltrating cells and molecular pathways in the PsA synovium.

Baseline synovial samples resembled previous descriptions of inflamed SpA synovial tissue with increased cellular infiltration and hypervascularity (22). Importantly, 12 weeks of ustekinumab treatment resulted in a numerical decrease of all infiltrating immune cells, except for plasma cells, and a significant decrease in CD68-positive sublining macrophages and MMP3 levels, both previously identified biomarkers for treatment response in SpA (12, 17, 23). In line with earlier studies investigating the effects of TNF and IL-17A inhibition (15, 16, 22), IL-12p40/IL-23p40 inhibition decreased PsA synovial inflammation, but did not normalize synovial histology. A persistent synovial cellular infiltrate can be observed in PsA patients in both clinical and ultrasound remission after effective treatment (22). Consequently, it could be hypothesized that either longer treatment is needed, or that targeting a single inflammatory molecule suffices to reduce clinical inflammation in PsA, but fails to completely reverse the complex disease pathophysiology on the cellular and molecular level. Frequent relapse of SpA after treatment cessation pleads for the latter (24–26).

Comparing synovial biopsies before and after different targeted treatments on the molecular level may enlighten us on (novel) pathways that are mainly IL-23/IL-17 or TNF driven, and on processes that remain active in clinically quiescent joints, as well as in patients with persistent disease activity after treatment. mRNA expression of IL-17A, IL-17F, and TNF, key cytokines of the two major pathways driving PsA/SpA disease, seemed unaffected by IL-12p40/IL-23p40 blockade with ustekinumab in our cohort. Previously, we have shown that synovial TNF expression is also unaffected by anti-IL-17A treatment (15), indicating that the IL-23/IL-17 pathway does not regulate the TNF pathway in the synovium. This may explain the differential effects of these treatments in individual patients. Since IL-23 is thought to be upstream of IL-17, reduced IL-17 expression after ustekinumab treatment would have been likely and was seen in psoriatic skin (27). Perhaps our study population was too small to observe a decline on the group level, as IL-17A expression did decrease in some patients. But besides IL-17A and IL-17F levels, levels of downstream cytokines IL-6 and IL-8 also did not decrease on the group level after ustekinumab treatment. A possible explanation for the lack of effect on IL-17A and F expression levels observed in our study could be that IL-17 can not only be produced in a IL-23-dependent fashion by Th17 cells, but also independent of IL-23 by innate like lymphocytes (28, 29), and that these IL-23 independent sources are more important in psoriatic synovium than skin. Unfortunately, we were unable to perform functional studies for verification. Similarly, anti-IL-23R treatment did not affect IL-17A/F expression in a therapeutic setting in an experimental SpA rat model (30). So our results might underline that the IL-23/IL-17 pathway does not function purely linear (31, 32), and that IL-12p40/IL-23p40 blockade exerts clinical effect through other ways than through modulating IL-17 levels.

The IL-12 cytokine family is postulated to have feedback mechanisms (1). However, we did not observe consistent changes as blocking of p40 only decreased IL-23p19 mRNA in the synovium, which suggests that cells producing IL-23, but not IL-12, may be affected by ustekinumab treatment. IL-23 is mainly produced by activated antigen-presenting cells, like dendritic cells and macrophages (33, 34), and macrophages were indeed decreased in our cohort. Yet, as we assessed mRNA expression levels in whole tissue, further *in vitro* studies are required to analyze and confirm which individual cells producing IL-23 are specifically affected.

To better understand the discrepancy between the histologic improvement and the apparent absence of impact on the IL-23/IL-17 and TNF pathways, we performed an unbiased RNAseq analysis of whole tissue followed by GO terms and KEGG pathway enrichment analyses to investigate whether p40 blockade affects other pathways in PsA synovitis. GO terms showed involvement of various pathways including chemotaxis and angiogenesis, two known prominent features in inflamed synovial tissue in SpA (22, 35, 36) in the downregulated DEGs. Specifically, gene expression of MS4A1, encoding CD20 expressed by B cells, and of CXCL6, involved in the attraction of neutrophils, were downregulated after ustekinumab treatment based on RNAseq and qPCR analysis, and numerical (albeit no significant) reductions of CD20+ B cells and CD15+ neutrophils were seen by IHC. Pro-angiogenic VEGFC and placental growth factor (PGF) were downregulated after ustekinumab treatment in RNAseq and confirmed for VEGFC by qPCR; the anti-angiogenic factor angiopoietin-like 1 (ANGPTL1) was upregulated in ACR20 responders in RNAseq and qPCR. However, expression of the MUC1 gene, which is upregulated in hypoxia and involved in hypoxia-induced angiogenesis (37), was increased after ustekinumab treatment. IHC staining for vWF showed a small but non-significant reduction. TNF blockade also affects synovial angiogenesis (17, 22, 38). Whereas, previously, we observed that synovial vWF was unaffected by IL-17 blockade (15).

Besides cellular infiltration and angiogenesis, pathway analysis also revealed modulation of (myo)fibroblasts and osteoblasts, and involvement of the Wnt, PI3K-Akt-mTOR and MAPK-ERK signaling pathways.

A clear impact of ustekinumab on fibroblast and myofibroblast pathways can be seen through enrichment of GO terms fibroblast growth factor receptor signaling pathway, positive regulation of fibroblast proliferation, and negative regulators of myofibroblast differentiation, together with decreased gene and mRNA expression of several FGFs. As we previously reported that SpA synovium has a myofibroblast gene signature compared to RA synovium (39), and here we show that myofibroblasts are affected after treatment, it will be interesting to further investigate the role of myofibroblasts in PsA pathology and to see how p40 blockade specifically affects PsA synovial fibroblasts in future studies.

The effects of p40 blockade on the Wnt signaling pathway and osteoblast differentiation is a very interesting finding, since the IL-23/IL-17 pathway and Wnt pathway are intimately involved in SpA bone pathology [reviewed in (40)]. Previously, we found multiple genes involved in Wnt signaling to be highly expressed in SpA compared to RA synovium (39). Genes of the Wnt signaling pathway were also differentially expressed when comparing PsA to healthy synovium (41). The ligand WNT9A, here upregulated after ustekinumab treatment, suppresses chondrogenesis and is an important factor for joint maintenance (42). Moreover, ustekinumab treatment significantly increased mRNA expression of CREB3L1 gene encoding a transcription factor involved in bone formation (43); CREB3L1 deficiency can cause severe osteogenesis imperfecta (44). Animal studies already suggested a role for IL-12p40 in bone remodeling, since IL-12p40 depletion in mice stimulated bone regeneration and increased bone mass, while IL-12p35 depletion (solely targeting IL-12) impaired bone regeneration and increased bone loss (45). Clinically, ustekinumab-treated PsA patients had significantly lower radiographic progression (i.e., lower Sharp/van der Heijde score on joint space narrowing and erosions) than the placebo-treated group (10). Though we assessed the synovial response, not the bone response to ustekinumab, our results collide with literature that ustekinumab might affect bone remodeling pathways in PsA and should be further investigated as systemic bone loss and local new bone formation remain major unmet needs in PsA and SpA management.

Several GO terms and the KEGG pathway for the PI3K-Akt-mTOR signaling pathway were overrepresented in the total group and ACR20 responders. Since the PI3K-Akt-mTOR signaling pathway can regulate Th17 cell differentiation (46), it is considered an interesting treatment target for SpA. We previously showed that the PI3K-AKT-mTOR signaling pathway is activated in SpA synovium and selective inhibition of PI3Kδ reduced the inflammatory response of immune cells, and skin and synovial fibroblasts in SpA (47). Inhibition of PI3Ks was also effective in murine psoriasis (48) and collagen-induced arthritis models (49). mTOR blockade with rapamycin inhibited arthritis and affected bone remodeling in a SpA rat model and also inhibited osteogenic differentiation of SpA patients' synovial fibroblasts *in vitro* (50). Based on the recent data from our team and from others (46–50), we expect that the observed modulation of the PI3K-Akt-mTOR pathway might take place by innate-like (MAIT and γδ-T cells) and adaptive (Th17) cells or synovial fibroblasts, although this remains to be formally tested in future studies. The potential effects of ustekinumab on the PI3K-Akt-mTOR pathway in our study supports the concept that specific targeting of the PI3K-Akt-mTOR pathway could be beneficial in human SpA.

The intricate mitogen-activated protein kinase (MAPK) signaling network transduces intercellular signals from the cell membrane to the three MAPK families of extracellular-signal-regulated kinase (ERK), c-Jun N-terminal kinase (JNK) and p38, which regulate gene expression important for cell proliferation, differentiation, survival, and apoptosis (51). The MAPK signaling pathway and ERK1 and ERK2 cascade were modulated by ustekinumab in either the total group or ACR20 responders. Genes of the MAPK signaling pathway are differentially expressed in PsA vs. healthy synovium (41). Activated MAPKs are present in PsA synovium and TNF blockade reduces activation of ERK and JNK, but not of p38 (52). Previously, we found active MAPKs expression in the synovium of early SpA patients, but lower than in early RA patients for ERK and JNK (53). Even though post-transcriptional modification regulates MAPK activation, our results reveal that ustekinumab modulates the MAPK-ERK signaling pathway at the transcriptional level, which might aid in controlling PsA disease. Ustekinumab thus seems to affect various inflammatory molecular pathways in the PsA synovium. Future studies may show how p40 blockade directly modulates these pathways.

In a *post-hoc* exploratory analysis, ACR20 responders and non-responders differed strikingly in their molecular response to ustekinumab in an unbiased analysis, namely multidimensional scaling of the total transcriptome. We could validate differential expression between the groups for three out of six genes by qPCR, namely ANGPTL1, NOV and AZIN1. ANGPTL1, upregulated in responders, suppresses the PI3K-Akt and MAPK-ERK signaling pathways and inhibits angiogenesis (54, 55). Expression of NOV, encoding a small secreted regulatory protein, was upregulated in responders; NOV overexpression attenuates the PI3K-Akt-mTOR pathway and decreases MMP production (56), while mice without NOV protein display an osteoarthritis-like disease (57). AZIN1 was downregulated in responders; AZIN1 promotes polyamine production, essential for cell growth, and differentiation, but AZIN1 overactivation can increase the invasive potential of fibroblasts (58) and can lead to cancer, inflammation or diabetes (59). Since qPCR only analyzes one mRNA variant and not all RNAs like RNAseq, it is difficult to say for the unconfirmed genes, whether the RNAseq was false positive or the qPCR analysis was too narrow. Although we could observe a difference despite our small sample sizes, 4 vs. 3 patients in RNAseq, respectively, because of our small sample sizes, we cannot draw firm conclusions. Confirmation in larger cohorts may allow us to find biomarkers, i.e., differential expression of specific genes at baseline, which may predict individual treatment response to ustekinumab.

To our knowledge, this is the first study to assess the effects of ustekinumab on the cellular infiltrate and molecular pathways in synovial tissue biopsies taken from PsA patients before and after treatment. Whole tissue RNA sequencing enabled us to see the overall synovial tissue response to IL-12p40/IL-23p40 blockade, but kept us from the effector cells of the observed response. RNA sequencing at the single-cell level, multiplexed spatial imaging of the synovial tissue and flow cytometric assays of freshly isolated synovial cells before/after the treatment combined with *in vitro* analyses establishing the direct effect of ustekinumab on IL-17-producing cells would further elucidate how the IL-23/IL-17 pathway contributes to PsA pathophysiology. Nevertheless, these analyses were out of the scope of the current study. Though joint counts improved for the majority of patients in our study, we observed only limited joint responses on the group level due to the nature of our study population: a rather small group of mostly oligoarthritic patients. However, our study population resembles the general PsA population since patients visiting the outpatient clinic more often have oligoarticular than polyarticular disease. We observed only numerical differences for most cellular markers, presumably due to the small sample size. Given our small sample size of only male participants, our results should be confirmed in larger cohorts containing both sexes.

Comparing our RNAseq data to the molecular effects of TNF and/or IL-17A inhibition might discriminate treatment-specific effects from general effects of reduced inflammation in the synovial tissue. If these treatment-specific effects complement each other—for instance targeting both inflammation and bone remodeling -, combined therapy approaches may be explored in PsA and SpA in the future.

Taken together, this mechanism of action study indicates that blocking of the p40 subunit shared by IL-12 and IL-23 by ustekinumab suppresses synovial inflammation in PsA through modulation of MAPK-ERK, Wnt and potentially also PI3K-Akt-mTOR signaling pathways rather than by directly impacting the IL-17 pathway. It also implies that certain PsA patients might benefit more from this treatment than others, although this has to be verified and confirmed on larger cohorts.

## Data Availability Statement

The datasets presented in this study can be found in online repositories. The names of the repository/repositories and accession number(s) can be found below: https://www.ncbi.nlm.nih.gov/, PRJNA693312.

## Ethics Statement

The studies involving human participants were reviewed and approved by The Medical Ethics Committee of the Academic Medical Center Amsterdam. The patients/participants provided their written informed consent to participate in this study.

## Author Contributions

All authors listed have made a substantial, direct and intellectual contribution to the work, and approved it for publication.

## Conflict of Interest

DB is currently an employee of UCB pharma. MS received consultancy fees from Novartis, Abvie, Eli Lilly and MSD, and research grants from Novartis, UCB and Eli Lilly. The remaining authors declare that the research was conducted in the absence of any commercial or financial relationships that could be construed as a potential conflict of interest. The authors declare that this study received funding from Janssen Pharmaceutica. The funder was not involved in the study design, collection, analysis, interpretation of data, the writing of this article or the decision to submit it for publication. The handling editor declared a past co-authorship with the authors HJ, DB, NY, MS, and LM.
