# Supplementary figures and images for: IL-12p40/IL-23p40 Blockade With Ustekinumab Decreases the Synovial Inflammatory Infiltrate Through Modulation of Multiple Signaling Pathways Including MAPK-ERK and Wnt
Source: Front Immunol. 2021 Mar 4;12:611656. doi: 10.3389/fimmu.2021.611656 (PMC7971179; doi:10.3389/fimmu.2021.611656)

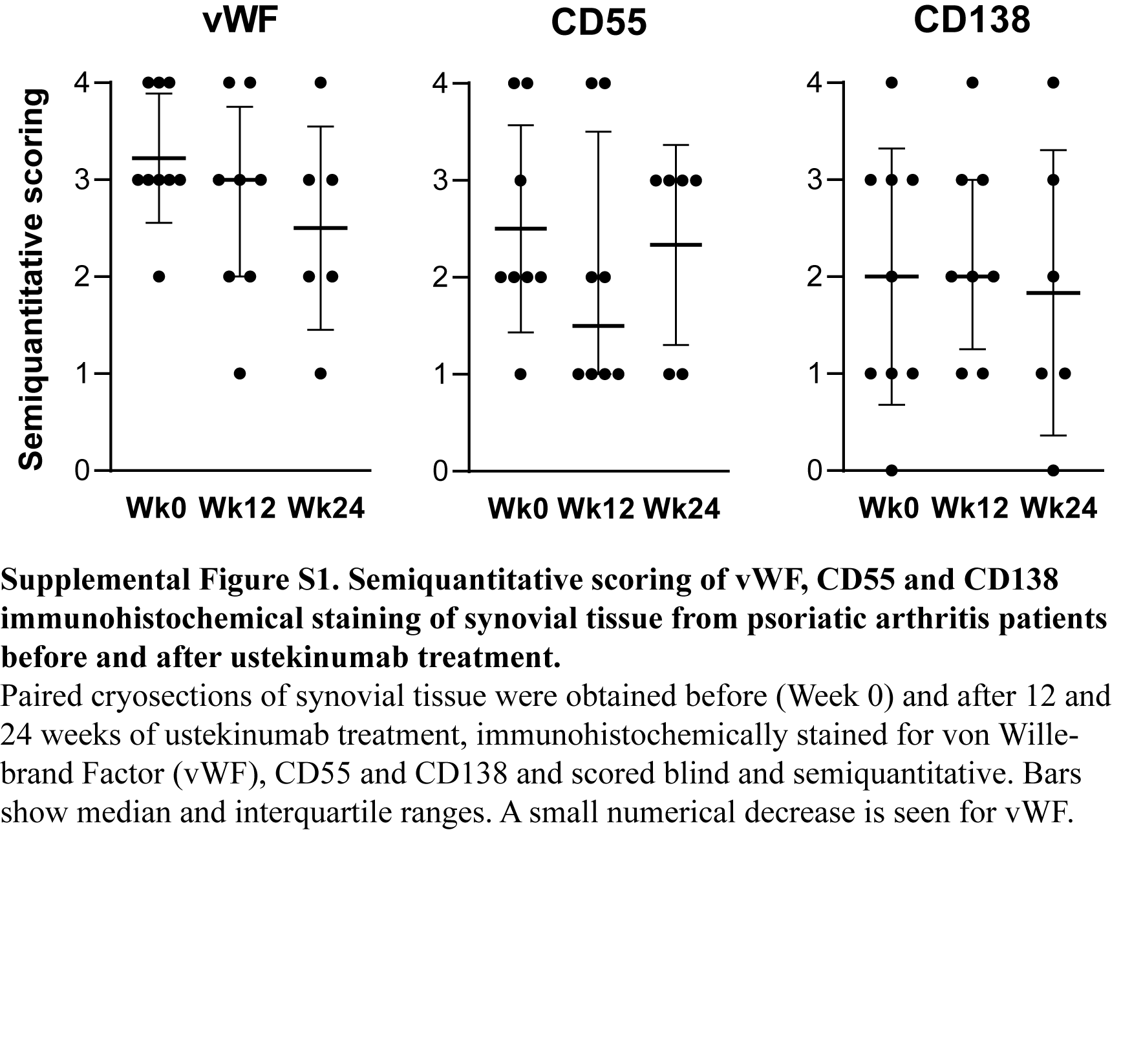

Supplement: Supplementary file 1 [file Image_1.TIF]
